# EmpPrior: using outside empirical data to inform branch-length priors for Bayesian phylogenetics

**DOI:** 10.1186/s12859-016-1132-4

**Published:** 2016-06-24

**Authors:** John J. Andersen, Bradley J. Nelson, Jeremy M. Brown

**Affiliations:** Department of Biological Sciences and Museum of Natural Science, Louisiana State University, 202 Life Sciences Building, Baton Rouge, LA 70803 USA; Present Address: IBM, 11501 Burnet Rd., Austin, TX 78758 USA; Present Address: Department of Genome Sciences, University of Washington, Foege Building S-250, Seattle, WA 98195 USA

**Keywords:** Bayesian phylogenetics, Informed priors, Branch lengths

## Abstract

**Background:**

Branch-length parameters are a central component of phylogenetic models and of intrinsic biological interest. Default branch-length priors in some Bayesian phylogenetic software can be unintentionally informative and lead to branch- and tree-length estimates that are unreasonable. Alternatively, priors may be uninformative, but lead to diffuse posterior estimates. Despite the widespread availability of relevant datasets from other groups, biologists rarely leverage outside information to specify branch-length priors that are specific to the analysis they are conducting.

**Results:**

We developed the software package EmpPrior to facilitate the collection and incorporation of relevant, outside information when setting branch-length priors for phylogenetics. EmpPrior efficiently queries TreeBASE to find data that are similar to focal data, in terms of taxonomic and genetic sampling, and uses them to inform branch-length priors for the focal analysis. EmpPrior consists of two components: EmpPrior-search, written in Java to query TreeBASE, and EmpPrior-fit, written in R to parameterize branch-length distributions. In an example analysis, we show how the use of relevant, outside data is made possible by EmpPrior and improves tree-length estimates from a focal dataset.

**Conclusion:**

EmpPrior is easy to use, fast, and improves both the accuracy and precision of branch-length estimates in many circumstances. While EmpPrior’s focus is on branch lengths, the strategy it employs could easily be extended to address other prior parameterization problems in phylogenetics.

## Background

Default branch-length priors for Bayesian phylogenetic analyses can lead to branch- and tree-length estimates that are incongruent with maximum-likelihood estimates (MLEs) [1–3]. Brown *et al.* [1] and Rannala *et al.* [3] investigated this issue and hypothesized that for these datasets a poorly specified prior can favor unreasonably long branch lengths and/or cause branch-length mixing problems. This behavior is troubling, because default priors are often thought to exert minimal influence on posterior estimates. In practice, the specification of non-informative priors may be essentially impossible for many parameters in phylogenetics.

Brown *et al.* [1] suggested an empirical Bayesian approach to alleviate these problems, where prior distributions are parameterized using focal data. While this method usually recovers ML tree lengths in resulting credible intervals, it has been criticized for being non-Bayesian and artificially reducing uncertainty. Rannala *et al.* [3] proposed, and Zhang *et al.* [4] evaluated, the use of a compound Dirichlet prior on branch- and tree-lengths that generally improves upon the default exponential branch-length prior. However, default settings did not recover the ML estimate for all datasets examined, which is problematic for non-informative priors.

For many phylogenetic questions, previously collected datasets are available that use the same genes and sample similar numbers of taxa in other clades. These outside data can be leveraged to inform prior distributions for analysis of focal data sets. Some previous work has attempted to incorporate outside molecular data into phylogenetic analyses in a principled manner [5], but exploration has been limited and has employed hierarchical modeling strategies not currently available in popular Bayesian phylogenetic software packages (e.g., MrBayes [6] and BEAST [7]).

**Fig. 1:**
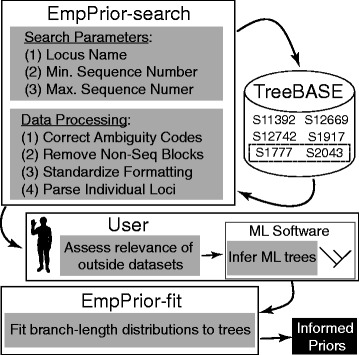
Flowchart for generating informed branch-length priors with EmpPrior. EmpPrior-search queries TreeBASE to find data similar to the focal data. Outside data are then used as input for maximum-likelihood (ML) tree searches. Branch-length distributions are fit to ML trees in EmpPrior-fit and parameter estimates are used to set priors for analysis of the focal data

The use of outside data to parameterize prior distributions in a focal analysis is relatively straightforward in principle (Fig. 1), but several procedural challenges arise in practice. First, available phylogenetic databases (e.g., TreeBase and Dryad) can be difficult to query, especially according to the criteria that might be important for establishing relevance of an outside dataset to a focal dataset (e.g., the number of taxa sampled). Also, the inclusion of branch-length information is uneven across deposited trees. Branch lengths might be missing entirely, they might have been estimated using a concatenated dataset, or an inappropriate method of inference might have been used. Second, once relevant trees with branch lengths have been obtained, no software exists to estimate relevant parameter values for the branch-length distributions that are used as priors in most Bayesian phylogenetic software. To alleviate these procedural challenges, we developed EmpPrior.

Beyond the procedural challenges, the process of informing priors with relevant data also requires some judgement by the researcher. Specifically for branch lengths, relevant outside datasets should contain sequences with similar evolutionary properties to the focal dataset and should employ similar patterns of taxonomic sampling, both in the number of sequences included in analysis and in the distribution of their relatedness. EmpPrior does not obviate the need for judgement in these areas, nor should it. Establishing relevance requires expertise and serves as an opportunity for researchers to apply their knowledge of the taxa in question to improve the accuracy and precision of their results. Rather, EmpPrior serves as a toolkit to marry the available outside information contained in both existing datasets and researcher expertise.

## Implementation

We developed EmpPrior to streamline the process of finding relevant, outside datasets and using them to set informed priors on branch lengths in ways consistent with the available options in widely used Bayesian phylogenetic software packages. EmpPrior consists of two components: EmpPrior-search and EmpPrior-fit (Fig. 1). EmpPrior-search queries TreeBASE with a user-defined gene name (or other user-chosen search term) and restrictions on dataset size, then parses and formats returned Nexus files for ML phylogeny inference. EmpPrior-fit optimizes parameters of branch-length distributions using ML phylogenies from outside datasets. Resulting parameter estimates can be copied directly into branch-length priors in Bayesian phylogenetic software.

EmpPrior-search is written in Java, so it is platform independent and requires no external dependencies. Users can supply EmpPrior-search with a list of gene name synonyms and the program will search titles and abstracts in TreeBASE for these terms. In preliminary tests, searches of the title and abstract fields gave the best tradeoff in returning a reasonable number of datasets, while maintaining their relevance to the original search terms. Nexus files associated with relevant studies will be filtered according to user-supplied maximum and minimum values for the number of sequences. EmpPrior-search will then process data files by replacing TreeBASE ambiguity codes (e.g., {A,C}) with standard International Union of Pure and Applied Chemistry (IUPAC) codes (e.g., M), removing all Nexus blocks not associated with the alignment, and, if possible, excising the gene of interest from a concatenated dataset. Processed data files are ready to be used as input for any standard ML phylogeny inference program (e.g., Garli, RAxML, PhyML).

**Fig. 2:**
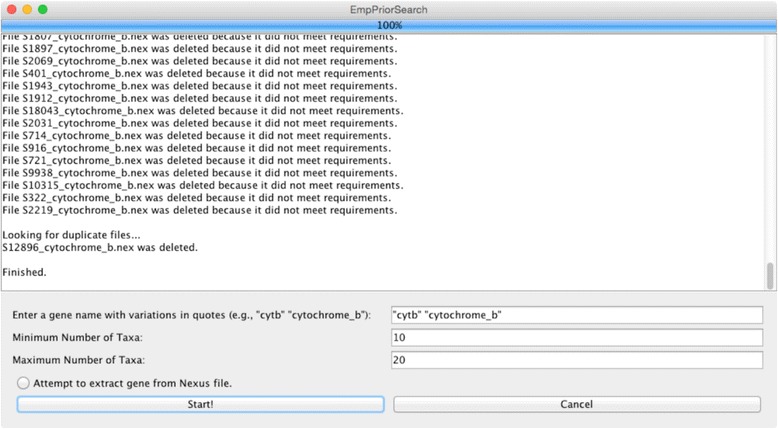
EmpPrior-search graphical user interface (GUI). The EmpPrior-search GUI allows users to specify the gene name and constraints on the number of taxa in a series of text fields at the bottom. These restrictions help to ensure that datasets returned from the search can provide relevant information to inform analysis of the focal data. A window in the middle of the GUI logs information about the progress of the TreeBase search and post-processing of datasets. A progress bar at the top provides users with a rough idea of EmpPrior-search’s progress. An optional post-processing step can be turned on with a radio button at the bottom, causing EmpPrior-search to attempt to extract the gene of interest from a multi-gene dataset. Due to inconsistencies in gene naming and data file formatting, this step can sometimes produce unreliable results. Users should always manually inspect relevant datasets to ensure that they have been parsed properly

EmpPrior-search utilizes the Java Swing class library to create a graphical user interface (GUI; Fig. 2) in which users can supply search terms to identify relevant datasets available in TreeBase. After identifying datasets with user-specified gene names in the titles and abstracts of TreeBase entries, EmpPrior-search uses independent threads to read each Nexus file from TreeBase using its corresponding uniform resource locator (URL). After downloading, the number of taxa in each data file is compared to user specifications and datasets not in the acceptable range are deleted. Remaining files are checked for duplicate Study IDs, in case the same dataset was returned by searches with different gene name synonyms, and duplicates are deleted. Datasets are then renamed to indicate both Study ID and the gene name by which they were identified. In the case of duplicates, only the first gene synonym in the list provided by the user will be used. In situations where titles and abstracts frequently contain multiple gene name synonyms, many more datasets will be downloaded than will be kept after post-processing. Nonetheless, EmpPrior-search usually only takes a few minutes to complete the process of downloading and post-processing, even when using multiple synonyms for popular genes. After file download and processing with EmpPrior-search, the user will need to make decisions about which datasets are relevant to the focal dataset and then perform ML phylogenetic inference using their preferred software.

EmpPrior-fit is written in R, making both components of the software platform independent. EmpPrior-fit harvests branch lengths from ML phylogenies inferred with outside datasets (from any source) and provides ML parameter estimates for common branch-length prior distributions (e.g., the exponential and compound Dirichlet). To read trees, EmpPrior-fit employs the R package ape [8] and to perform ML parameter estimation it uses the bbmle package [9]. Probability densities (i.e., likelihoods) for simple distributions (e.g., the exponential) employ R’s built-in functionality. Likelihoods for the compound Dirichlet are calculated according to equation 36 from Rannala *et al.* [3]. Likelihoods are reported by EmpPrior-fit, although we caution that better fit of a particular distribution to the branch lengths estimated from an outside dataset does not necessarily mean that the same distribution has desirable properties as a prior for the analysis of a focal dataset. Other potential distributions for branch lengths can easily be added to EmpPrior-fit, should they be of interest or become available in future implementations of Bayesian phylogenetic software. All of the calculations implemented in EmpPrior-fit are fast, so even large tree sets can be rapidly processed with modest computing resources.

## Results and discussion

We ran EmpPrior-search on three gene names commonly found in phylogeographic studies (cytochrome b [using “cytb”, “cyt_b”, “cytochromeb”, and “cytochrome_b”], cytochrome oxidase I [using “COI” and “cytochrome_oxidase_I”], and ribulose-bisphosphate carboxylase [using “rbcL”]), because analyses of datasets consisting mainly of intraspecific samples seem particularly sensitive to the chosen branch-length prior. The three runs were performed on a 2.8 GHz Intel Core i7 MacBook Pro laptop and took 19 s (returning 59 datasets), 134 s (returning 93 datasets), and 135 s (returning 111 datasets), respectively.

The time required to infer ML trees from outside datasets will depend on the size of the dataset and the chosen software, but will always be the slowest step in the informed prior procedure as we have framed it here. Conveniently, the ML trees that we use to fit branch-length distributions do not require support values. By avoiding the need to bootstrap, or estimate posterior probabilities, for outside datasets the time required to infer ML trees is much faster than it would be for a standard empirical analysis. However, our preliminary analyses do suggest that thorough, model-based inference of branch lengths is necessary to find fitted parameter estimates that perform well in focal analyses.

**Fig. 3:**
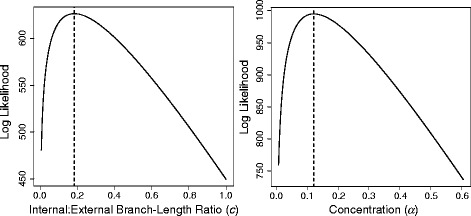
Log-likelihood surfaces for *c* and *α* of the compound Dirichlet branch-length distribution. Both log-likelihood surfaces were calculated using maximum-likelihood (ML) branch lengths based on a dataset of cytochrome *b* and 16S sequences from alpine newts (*Mesotriton alpestris*) with TreeBase Study ID S1777 [11]. The left plot shows log-likelihoods based on the compound Dirichlet distribution [3] for different values of the internal:external branch-length ratio (*c*) with all other parameters fixed. The right plot shows log-likelihoods for different values of the concentration parameter (*α*) with all other parameters fixed. The dashed line in each plot shows the ML estimate for each parameter returned by EmpPrior-fit

Fitting branch-length distributions to ML trees with EmpPrior-fit is very fast (<1 s per tree) and adds only a trivial amount of time when compared with ML tree inference. Likelihood surfaces are often smooth and unimodal for the parameters of interest (Fig. 3), so EmpPrior-fit is able to robustly find maximum-likelihood estimates. To our knowledge, no other software is designed to specifically estimate the parameters of branch-length distributions, although the numerical capabilities certainly exist in other programming languages and platforms.

Some professional judgement will always be necessary to choose which of the datasets returned from EmpPrior-search would be most appropriate for informing priors used in the analysis of a focal dataset. In the context of a Bayesian analysis, the prior serves as the point where such judgement can naturally and appropriately be exercised. We are of the opinion that researchers are better equipped to judge the relevance of outside datasets that are then used to parameterize branch-length distributions, than they are to judge the appropriateness of parameter estimates directly. This difference in judgement ability may become more pronounced as preferred branch-length priors become more complex (e.g., the compound Dirichlet) [3, 4] and employ distributions less familiar to many biologists (e.g., the Dirichlet). By exercising judgement in the choice of outside datasets, and then fitting relevant distributions to those data, our approach allows researchers to specify an informed prior in a principled manner.

To demonstrate the utility of informed priors parameterized using the approach we outline, EmpPrior was used to set informed branch-length priors for a dataset of 65 mtDNA sequences (COII and 16S) from the brittle star, *Astrotoma agassizii*. This dataset has previously been shown to return spurious tree-length estimates under the default exponential branch-length prior in MrBayes [2]. MrBayes 3.2.2 [6] was used to estimate tree length under both default and informed parameterizations of the exponential and compound Dirichlet [3] branch-length priors. Informed parameter estimates were based on two relevant outside datasets, each containing one of the genes in the focal dataset (COII: S2043, 16S: S1777). These datasets had a depth and pattern of sampling (intraspecific) similar to the focal data. Informed parameter values were estimated for both the exponential and compound Dirichlet distributions (with *α* and *c* estimated both singly and jointly for the compound Dirichlet). For both prior distributions and both outside datasets, tree-length estimates from informed priors were closer to the MLEs than default estimates (Table 1). For compound Dirichlet priors with informed values of *α*, 95 % highest posterior density intervals of tree length included the MLE (Table 1). Elsewhere, we describe more widespread testing of the informed prior approach and discuss its strengths and limitations [10].

**Table 1:** Default and informed tree-length estimates

Outside Dataset	*λ*	*α*	*c*	MLE	95 % HPD
None (default)	10			0.40	[10.54–14.94]
		1	1	0.40	[0.24–0.34]
S1777	95.6			0.40	[0.83–1.25]
		**0.12**	**1**	0.40	**[0.30–0.48]**
		1	0.18	0.40	[0.26–0.39]
		**0.11**	**1.35**	0.40	**[0.30–0.49]**
S2043	80.7			0.40	[1.03–1.53]
		**0.13**	**1**	0.40	**[0.30–0.49]**
		1	0.27	0.40	[0.26–0.38]
		**0.10**	**1.93**	0.40	**[0.29–0.46]**

Highest posterior density intervals (95 % HPDs) of tree length (TL) for analyses of a focal dataset from brittle stars [12]. Each individual row corresponds to a Bayesian analysis of the focal data with different prior settings for branch- and tree-lengths. We used both default and informed parameterizations of the exponential (*λ*) and compound Dirichlet (*α*, *c*) branch-length priors. The top set of two shaded rows show default settings and focal inferences. Below that, alternate shadings indicate prior settings parameterized with different outside datasets (S1777 and S2043). The focal TL MLE was produced with Garli [13]. Focal HPDs in bold contain the MLE

While the informed approach to setting branch-length priors that we describe has been applied primarily to datasets with a small number of loci, it may also be used for large datasets with similar levels of divergence across loci (e.g., ultraconserved elements). In fact, such large datasets allow for an even more direct application of informed priors, if a small subset of the data is used to inform priors that are then applied to analyses of the remaining data. Future work with EmpPrior will explore the performance of this approach for datasets generated by targeted sequence capture. Increases in the number of publicly available multiple sequence alignments, generated both through Sanger and high-throughput sequencing methods, will facilitate more widespread use of informed priors for branch lengths and other parameters of interest.

## Conclusions

By using outside datasets with sampling depths (e.g., species-level) and evolutionary properties that are similar to a focal dataset, researchers can avoid the circularity of empirical Bayesian approaches and improve upon default prior performance [10]. Despite the appeal of this approach, several procedural hurdles make it difficult to apply in practice. We developed EmpPrior to help overcome these hurdles and have shown that it can improve the accuracy and precision of branch-length estimates when compared to the default settings of popular Bayesian phylogenetic software. We hope that informed priors see more widespread use in Bayesian phylogenetics.

## Availability and requirements

*Project name:* EmpPrior

*Project home page:*https://github.com/jembrown/EmpPrior

*Operating systems:* Platform independent

*Programming language*: Java and R

*Other requirements:* EmpPrior-search: Java version 1.8; EmpPrior-fit: R version 3.2, CRAN packages ape and bbmle.

*License*: GNU GPL v3

*Any restrictions to use by non-academics*: None

## Abbreviations

GUI, graphical user interface; IUPAC, International Union of Pure and Applied Chemistry; ML, maximum likelihood; MLE, maximum-likelihood estimate; URL, uniform resource locator.
